# A Meta‐Analysis of the Association Between Early Venous Filling and Hemorrhagic Transformation After Endovascular Treatment in Acute Large Vessel Occlusion

**DOI:** 10.1002/brb3.70663

**Published:** 2025-07-07

**Authors:** Jiayu You, Xingqiang Li

**Affiliations:** ^1^ Department of Neurology and Neuroscience, Shenyang First People's Hospital Shenyang Brain Institute Shenyang Liaoning China; ^2^ Department of Neurology the Fourth Affiliated Hospital of China Medical University Shenyang China

**Keywords:** early venous filling, endovascular treatment, hemorrhagic transformation, meta‐analysis, poor prognosis

## Abstract

**Objective:**

This meta‐analysis assessed the link between early venous filling (EVF) and hemorrhagic transformation (HT) post‐endovascular treatment (EVT) in acute ischemic stroke (AIS) patients with large vessel occlusion (LVO).

**Materials and Methods:**

We searched PubMed, Embase, and Cochrane Library. Two reviewers independently screened studies, extracted data, and selected articles per preset criteria. The quality of included studies was assessed by the Newcastle–Ottawa Scale, and RevMan 5 was used for meta‐analysis. Stata 15 was used to test for publication bias.

**Results:**

Eight studies with 1758 AIS patients were included. EVF was associated with a higher HT rate after EVT (OR 4.5, 95% CI 3.47–5.85, *p* < 0.001). Subgroup analyses showed EVF was linked to higher incidences of hemorrhagic infarction (HI; OR 1.66, 95% CI 1.03–2.67, *p* = 0.04), parenchymal hematoma (PH; OR 3.86, 95% CI 2.55–5.83, *p* < 0.001), and symptomatic intracerebral hemorrhage (sICH; OR 5.95, 95% CI 2.99–11.83, *p* < 0.001). EVF was also related to a higher 90‐day poor prognosis rate (OR 3.12, 95% CI 2.14–4.54, *p* < 0.001).

**Conclusion:**

This meta‐analysis shows EVF positively correlates with HT and poor prognosis after EVT, having significant implications for acute LVO stroke management.

## Graphical abstract

In acute ischemic stroke patients caused by large vessel occlusion, early venous filling after endovascular treatment indicates a higher risk of hemorrhagic transformation and poor outcome. Adjust the treatment strategies timely may reduce the risk of symptomatic cerebral hemorrhage.

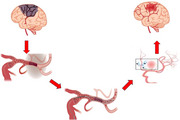

## 1 Introduction

Acute ischemic stroke (AIS) stands as one of the primary diseases responsible for human disability and mortality. Among them, large vessel occlusion (LVO) accounts for 30% of the total cases, characterized by a high rate of disability and mortality (Malhotra et al. 2017). Early and rapid reperfusion therapies, such as intravenous thrombolysis (IVT) and endovascular treatment (EVT), play a crucial role in effectively restoring blood flow to ischemic brain tissue. By salvaging the infarcted cerebral tissue, these therapies have the potential to improve the prognosis of patients.

However, IVT alone has a relatively low successful recanalization rate for AIS caused by acute LVO. Moreover, IVT is contraindicated in patients who have surpassed the therapeutic time window. In 2015, five randomized controlled trials demonstrated that EVT, either alone or in combination with IVT, can more effectively recanalize LVO within 6 h compared to IVT alone (Berkhemer et al. 2015; Goyal et al. 2015; Campbell et al. 2015; Jovin et al. 2015; Saver et al. 2015). Over the past decade, with the remarkable advancements in neuroimaging and recanalization techniques, EVT has emerged as an efficient treatment option for AIS due to LVO within the 6–24‐h time frame (Jovin et al. 2017; Albers et al. 2017). Clinical evidence has shown that EVT not only effectively reduces the mortality rate of AIS patients with LVO but also significantly enhances their long‐term prognosis (Goyal et al. 2015; Saver et al. 2015; Politi et al. 2017). Despite the fact that the majority of patients with acute LVO achieve successful recanalization after EVT, a substantial 43% of these patients still fail to achieve a favorable outcome, mainly due to hemorrhagic transformation (HT) and malignant cerebral edema (Hao et al. 2017; Huang et al. 2019). Given that HT can have a detrimental impact on the final prognosis, the ability to predict its occurrence at the earliest possible stage and promptly adjust treatment strategies is of utmost importance (Desai et al. 2020). In recent years, numerous studies have identified several indicators capable of predicting HT after EVT (Tian et al. 2022; Cannarsa et al. 2022; Zhang et al. 2021; Fritzsch et al. 2015; Han et al. 2025). Among these, the early appearance of local draining veins in the arterial ischemic area during the recanalization process, known as the early venous filling (EVF) sign, has drawn significant attention (Fritzsch et al. 2015; Han et al. 2025). With the continuous evolution of EVT technology, the presence of EVF during intraoperative digital subtraction angiography (DSA) has become a topic of great interest in the medical community.

The concept of EVF was first reported by Ferris et al. (1968) based on cerebral angiography. Subsequently, Ohta et al. (2004) discovered the appearance of EVF during angiography after intra‐arterial thrombolysis in AIS patients and hypothesized that EVF might serve as a predictive sign for parenchymal hematoma (PH). Cartmell et al. (2018) demonstrated a strong correlation between EVF and symptomatic parenchymal hemorrhage after EVT. Moreover, Shimonaga et al. (2020) identified EVF as a marker of hyperperfusion in patients with cardioembolic stroke after EVT and further revealed that EVF increases the risk of HT and unfavorable outcome. EVF, which is defined as the appearance of any cerebral vein prior to the late arterial phase, has been associated with the rapid passage of blood through the infarcted brain tissue and an increase in vascular permeability following the disruption of the blood–brain barrier. This phenomenon, also known as luxury perfusion, may potentially indicate the occurrence of infarct or HT after EVT (Liang et al. 2021). Based on the timing of venous filling, EVF can be classified into the arterial‐phase EVF and the capillary‐phase EVF. In addition, according to the pathway of venous filling, it can be categorized into two types: cortical artery‐to‐cortical vein (Type I) and lenticulostriate artery‐to‐thalamostriate vein (Type II). A previous study has suggested that Type II thalamostriate vein EVF is associated with a higher risk of symptomatic intracerebral hemorrhage (sICH) compared to Type I cortical vein EVF (Ohta et al. 2004).

Despite the existing research on the correlation between EVF and HT, most of these studies are single‐center, retrospective, and small‐sample cohort studies. The limited sample size in these studies reduces statistical power. Therefore, we used the existing evidence to perform a systematic review and meta‐analysis, which is expected to make some contributions to the field. Clinically, the findings will provide clinicians with a predictive indicator. This will enable doctors to assess the risk of HT in patients undergoing EVT, allowing for the development of more personalized treatment plans. In addition, the results of this study can serve as a reference for future large‐scale, multicenter, and prospective studies, promoting further progress in the treatment of AIS and ultimately improving patient outcomes.

## 2 Methods

This systematic review was carried out in accordance with the Preferred Reporting Items for Systematic reviews and Meta‐Analyses (PRISMA) statement and the Cochrane guideline for systematic reviews of interventions.

### 2.1 Inclusion Criteria

Studies that met the following criteria were included: (1) Human studies were considered with patients aged ≥ 18 years. (2) The time interval from the onset of symptoms to groin puncture should be ≤ 24 h, and no intracranial hemorrhage could be detected in the initial computed tomography (CT). (3) Patients diagnosed with AIS caused by LVO by any neuroradiological examination listed below (e.g., CT angiography [CTA], magnetic resonance angiography [MRA], or DSA). (4) Functional outcome measurements should be reported in the research either in different assessment tools (e.g., National Institutes of Health Stroke Scale [NIHSS], modified Rankin Scale [mRS]). A favorable prognosis was defined as an mRS score of 0–2 at 90 days. (5) Cross‐sectional studies, cohort studies, and case‐control studies will be involved in the review. (6) Papers concerning the epidemiology, clinical manifestations, treatment, or prognosis, especially with either logistic regression, Cox regression, or any inferential‐statistical analysis, were eligible. (7) The patients either received or were preparing to receive EVT (including retrievable stent thrombectomy, contact aspiration thrombectomy, arterial thrombolysis, inter‐arterial reperfusion, balloon angioplasty, stent angioplasty, etc.) or a combination of IVT and EVT as the primary treatment modality. (8) The presence of EVF was evaluated by DSA.

### 2.2 Exclusion Criteria

We excluded studies with the following criteria: (1) Studies that did not provide empirical data (e.g., reviews, commentaries, opinions, case reports, interviews, theoretical papers, conference abstracts, letters, and gray literature). (2) Studies with less than 10 stroke cases and without a comparison between the EVF and non‐EVF groups. (3) The full text was not available. (4) Studies without the rates of HT or sICH. The criteria for judging and classifying HT after EVT were mainly based on the European‐Australasian Acute Stroke Study II (ECASS II) (Larrue et al. 2001). sICH was defined as any PH, subarachnoid hemorrhage, or intraventricular hemorrhage accompanied by a ≥ 4‐point worsening of NIHSS score within 24 h.

### 2.3 Search Strategy

The search strategy was designed in accordance with the “Cochrane Guidelines for Systematic Reviews of Health Promotion and Public Health Interventions.” Prior to March 2025, we systematically scoured for English‐language articles in the PubMed, Embase, and Cochrane databases. The search strategy employed was as follows: ((endovascular treatment) OR (mechanical thrombectomy) OR (thrombectomy) OR (reperfusion)) AND ((early venous filling) OR (early cerebral vein) OR (early venous drainage)). The search strategies were adjusted according to the different databases (seen in the Supporting Information).

### 2.4 Study Selection

All studies from the selected databases were imported into EndNote 20 for duplication checking. Title/abstract screening was independently performed by two authors. Any discrepancies were resolved by a consultant with the corresponding author.

### 2.5 Data Extraction and Synthesis

A data extraction form was designed in the EXCEL, including Country, Study ID, Author/Year, Sample Size, Functional Outcome Measurement, and Prognostic Risk Factors. Data from full‐text articles were extracted by two authors independently.

### 2.6 Statistical Analysis

The data extracted from each study were analyzed using SPSS version 29.0 (IBM). More than three studies that reported the same outcome measures were included in the quantitative analysis; otherwise, a narrative approach was adopted. Continuous variables were extracted as mean and standard deviation (SD). The RevMan 5 software was employed for the meta‐analysis to calculate the odds ratio (OR) and 95% confidence interval (CI). The Cochrane *Q* test and *I* ^2^ statistic were used for heterogeneity testing. If *p* > 0.1 and *I* ^2^ < 50%, it indicates that there is no significant heterogeneity among the studies, and the fixed‐effect model was used to pool the effect sizes. Conversely, when there is heterogeneity among the studies, a random‐effects model was adopted. The output of *p* < 0.05 as statistically significant. Group analysis was then carried out to identify the source of heterogeneity, and sensitivity analysis was performed to assess the stability of the results. The publication bias is presented in the funnel plot.

### 2.7 Sensitivity Analyses

Sensitivity analyses were conducted by altering the effects model and using the leave‐one‐out method (omitting one trial each time and repeating the meta‐analysis) to further test the robustness of the findings.

### 2.8 Quality Analysis

We evaluated the quality of the included studies using the Newcastle–Ottawa scale (NOS) as a guideline. The cohort, case‐control, and cross‐sectional studies were all included in the NOS. This 9‐item checklist is designed to assess noncontrolled studies and provides an overall quality rating. For each item, a study can be assigned a maximum of one star based on whether the answer is “Yes,” “No,” or “Unclear” (Stang 2010). A study with a score of ≥ 6 is considered to be of high quality with a low risk of bias, while a score of < 5 is classified as low‐quality with a high inherent risk of bias. Stars were assigned to each observational category. All included studies were independently assessed by two authors, and any disagreements were decided by the corresponding author.

## 3 Results

**FIGURE 1:**
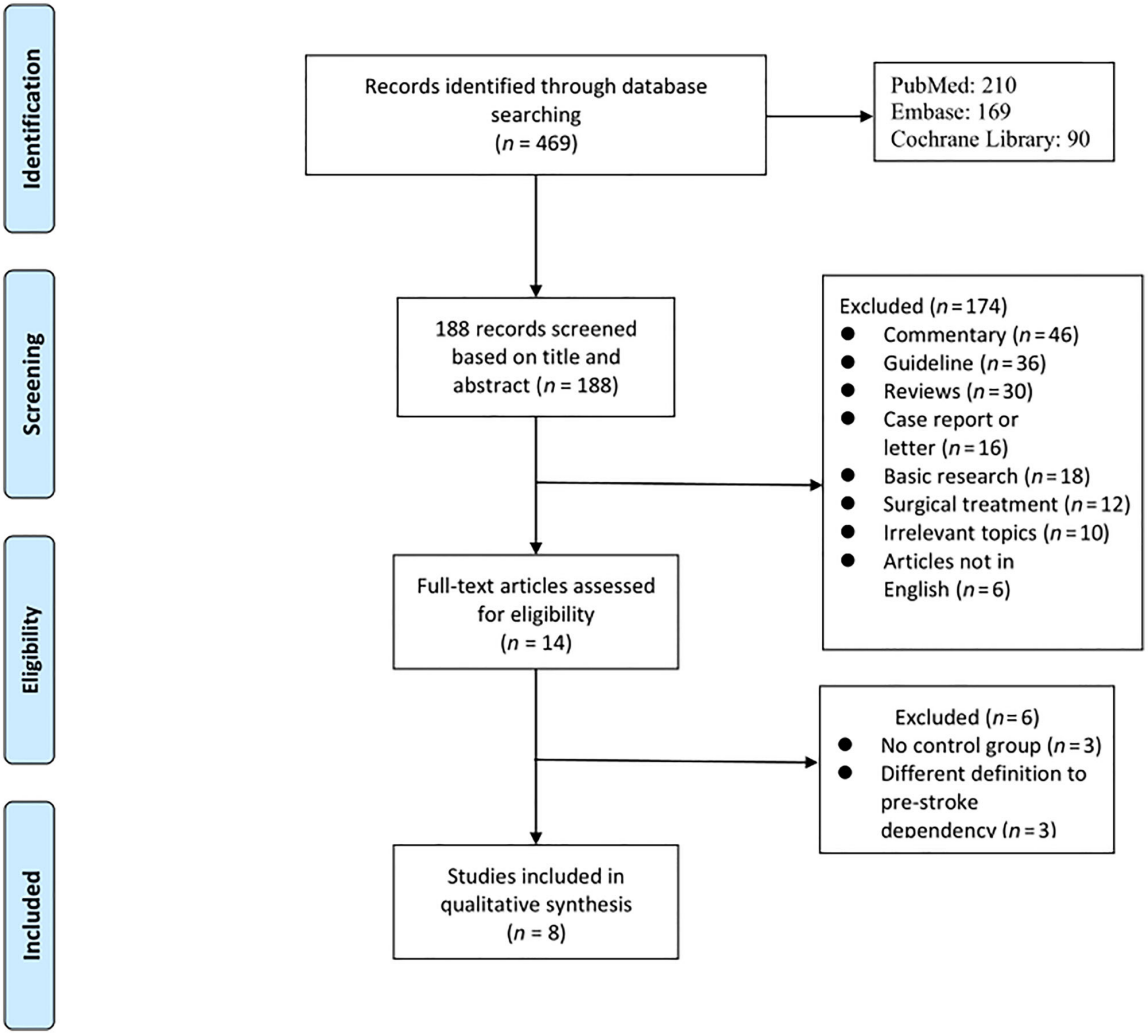
PRISMA flow diagram.

Based on the pre‐established inclusion criteria, a comprehensive search across three databases initially identified a total of 188 relevant studies. Subsequently, two independent authors carefully perused the full‐text articles. After a meticulous review of titles and abstracts, 174 citations were excluded, as illustrated in Figure 1.

The full text of 14 articles underwent in‐depth scrutiny, and ultimately, 8 studies involving 1758 patients were selected for inclusion in both the review and meta‐analysis (Table 1) (Han et al. 2025; Ohta et al. 2004; Cartmell et al. 2018; Shimonaga et al. 2020; Elands et al. 2021; Janvier et al. 2022; Yu et al. 2023; Li et al. 2024). These selected articles were meticulously analyzed, and the data were utilized for the meta‐analysis. The clinical data of the studies incorporated into the meta‐analysis are presented in Table 1. These studies were conducted between 2004 and 2025, originating from diverse geographical regions: France (1 study), Europe (1 study), China (3 studies), Japan (2 studies), and the United States (1 study). All the included studies in our analysis scored more than 5 points, with scores ranging from 7 to 9 (Table 2).

**TABLE 1:** Baseline characteristic description of the included studies.

Reference	Country	Study design	Participants	Males (%)	Operation	Primary endpoint	Secondary endpoint	Clinical follow‐up
Ohta et al. (2004)	Japan	A prospective cohort study	104	57.7	Inter‐arterial reperfusion	Hemorrhagic transformation	Favorable outcome	3 months
Cartmell et al. (2018)	USA	A retrospective cohort study	64	37.5	Endovascular treatment	Parenchymal hemorrhage	Mortality and favorable outcome	90 days
Shimonaga et al. (2020)	Japan	A prospective cohort study	35	43.0	Endovascular therapy	Hemorrhagic transformation	Hyperperfusion	90 days
Elands et al. (2021)	Germany	A retrospective cohort study	147	68.7	Thrombectomy	Reperfusion hemorrhage	Functional outcomes	90 days
Janvier et al. (2022)	France	A prospective cohort study	402	51.5	Mechanical thrombectomy	Symptomatic intracerebral hemorrhage	Favorable outcome	90 days
Yu et al. (2023)	China	A retrospective cohort study	350	54.8	Endovascular thrombectomy	Hemorrhagic transformation	Parenchymal hematoma	90 days
Li et al. (2024)	China	A retrospective cohort study	349	67.0	Mechanical thrombectomy	Intracerebral hemorrhage	Symptomatic intracerebral hemorrhage	90 days
Han et al. (2025)	China	A retrospective cohort study	307	60.9	Mechanical thrombectomy	Unfavorable outcomes	Hemorrhagic transformation	90 days

**TABLE 2:** Quality assessment of included studies by NOS.

Study ID	Year	Selection				Comparability		Exposure			Stars
		Q1	Q2	Q3	Q4	Q5	Q6	Q7	Q8	Q9	
Ohta et al.	2004	—	^*^	^*^	^*^	^*^	^*^	^*^	^*^	—	7
Cartmell et al.	2018	^*^	^*^	^*^	^*^	—	^*^	^*^	^*^	^*^	8
Shimonaga et al.	2020	^*^	^*^	^*^	^*^	^*^	^*^	—	^*^	^*^	8
Elands et al.	2021	^*^	^*^	^*^	^*^	—	^*^	^*^	^*^	^*^	8
Janvier et al.	2022	^*^	^*^	^*^	^*^	^*^	^*^	^*^	^*^	^*^	9
Yu et al.	2023	^*^	^*^	—	^*^	—	^*^	^*^	^*^	^*^	7
Li et al.	2024	^*^	^*^	^*^	^*^	^*^	—	^*^	^*^	^*^	8
Han et al.	2025	—	^*^	^*^	^*^	^*^	^*^	^*^	^*^	^*^	8

### 3.1 EVF Increases the Risk of HT

**FIGURE 2:**
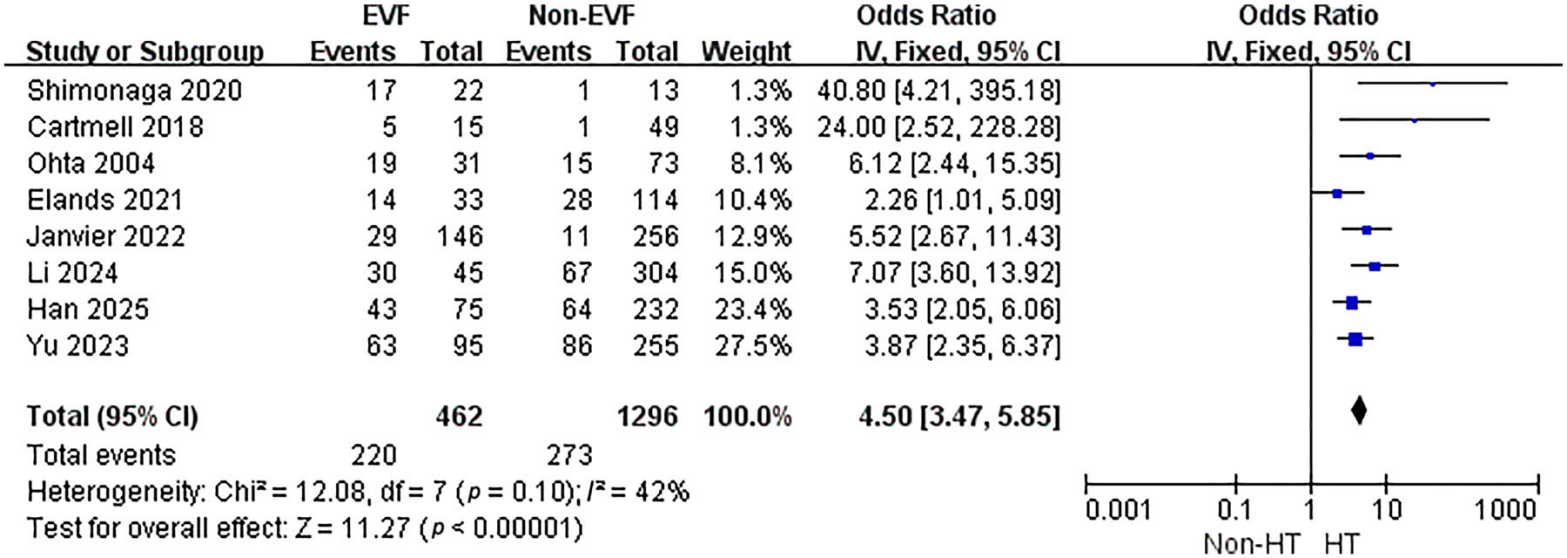
The association between EVF and HT.

A total of 8 studies were included in this particular analysis. To evaluate the association between EVF and HT subsequent to EVT, a meta‐analysis of these 8 studies was carried out. The results of the heterogeneity test revealed that there is no significant heterogeneity among the studies (*p* = 0.1, *I* ^2^ = 42%). Consequently, a fixed‐effect model was employed for the subsequent analysis (Figure 2). The findings demonstrated that the presence of EVF was associated with a higher rate of HT after EVT (OR = 4.5, 95% CI: 3.47–5.85, *p* < 0.001).

### 3.2 EVF Increases the Risk of HI

**FIGURE 3:**
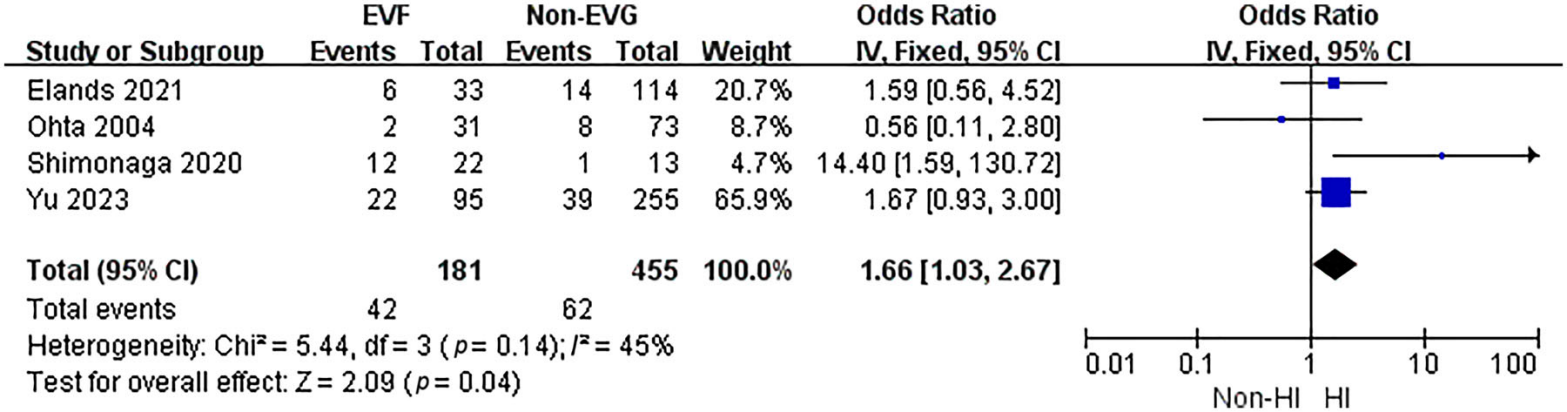
The association between EVF and HI.

In an effort to assess the relationship between EVF and HI after EVT, a meta‐analysis of 4 included studies was performed. The heterogeneity test indicated no significant heterogeneity among these studies (*p* = 0.14, *I* ^2^ = 45%). As a result, a fixed‐effect model was utilized for the analysis (Figure 3). The results clearly showed that the presence of EVF was associated with a higher incidence of HI after EVT (OR = 1.66, 95% CI: 1.03–2.67, *p* = 0.04).

### 3.3 EVF Increases the Risk of PH

**FIGURE 4:**
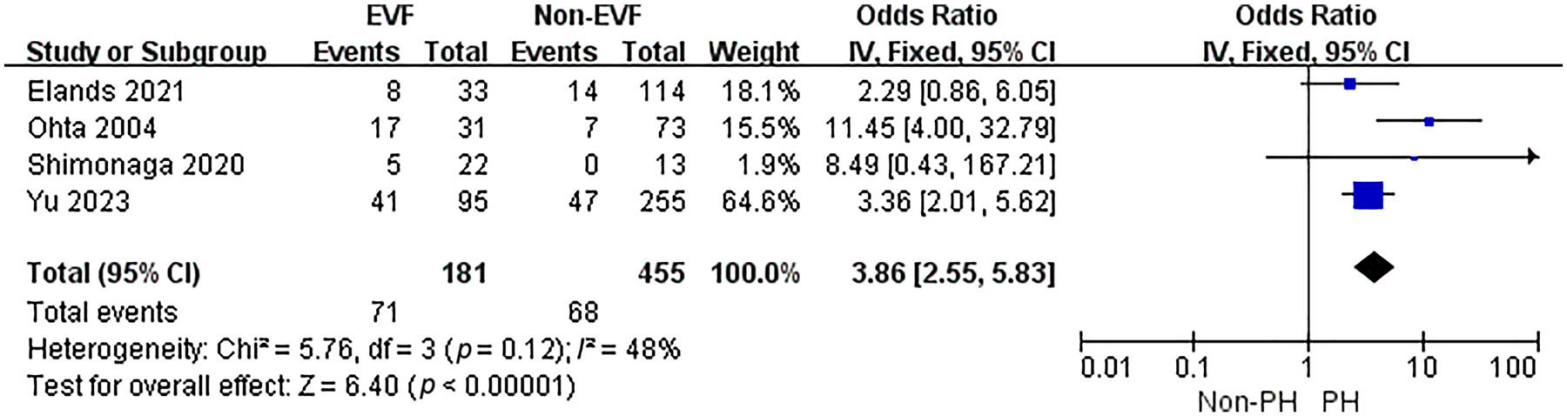
The association between EVF and PH.

To explore the connection between EVF and PH after EVT, a meta‐analysis of 4 included studies was conducted. The heterogeneity test results suggested no significant heterogeneity among the studies (*p* = 0.12, *I* ^2^ = 48%). Thus, a fixed‐effect model was adopted for the analysis (Figure 4). The outcomes indicated that the presence of EVF was linked to a higher rate of PH after EVT (OR = 3.86, 95% CI: 2.55–5.83, *p* < 0.001).

### 3.4 EVF Increases the Risk of sICH

**FIGURE 5:**
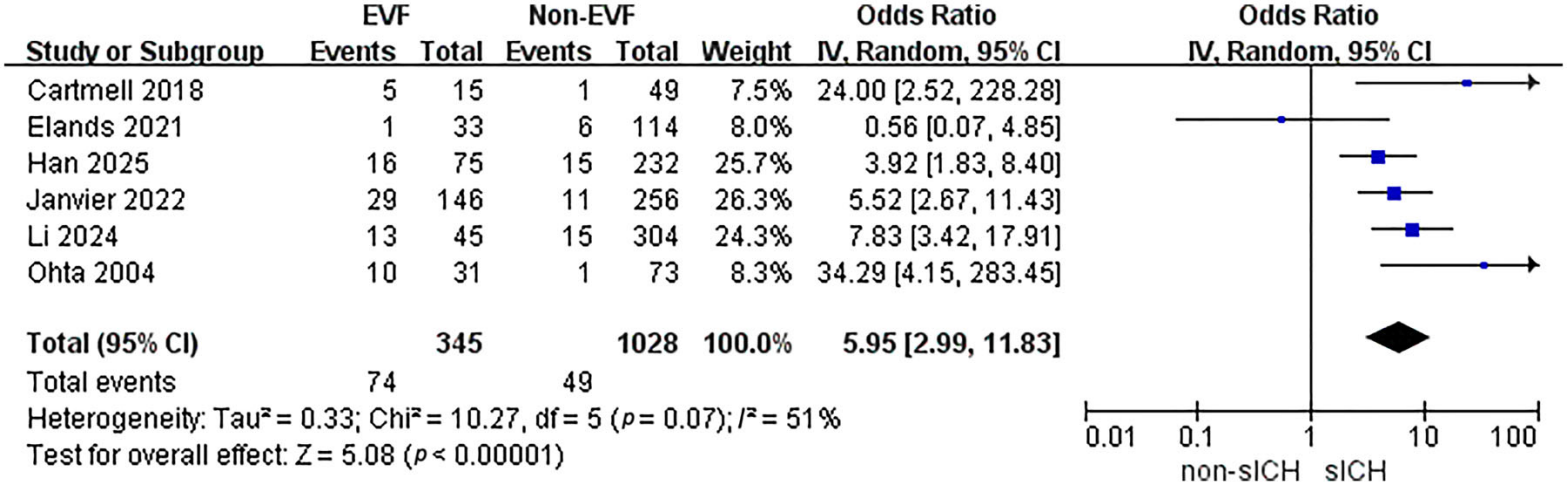
The association between EVF and sICH.

When assessing the association between EVF and sICH after EVT, a meta‐analysis of 6 included studies was carried out. The heterogeneity test detected significant heterogeneity among the studies (*p* = 0.07, *I* ^2^ = 51%). Therefore, a random‐effects model was utilized for the analysis (Figure 5). The results indicated that the presence of EVF was associated with a higher rate of sICH after EVT (OR = 5.95, 95% CI: 2.99–11.83, *p* < 0.001). The heterogeneity among studies was detected, which might be linked to potential fluctuations caused by insufficient statistical precision in studies with smaller sample sizes. Although the statistical evidence for heterogeneity was close to significance, the *I* ^2^ value indicated the presence of actual differences. Therefore, a random‐effects model was employed to conservatively estimate the effect size. Prospective studies are warranted in the future to standardize operational definitions and outcome evaluation criteria, thereby further validating the robustness of this conclusion.

### 3.5 EVF Increases the Risk of Poor Prognosis (mRS > 2)

**FIGURE 6:**
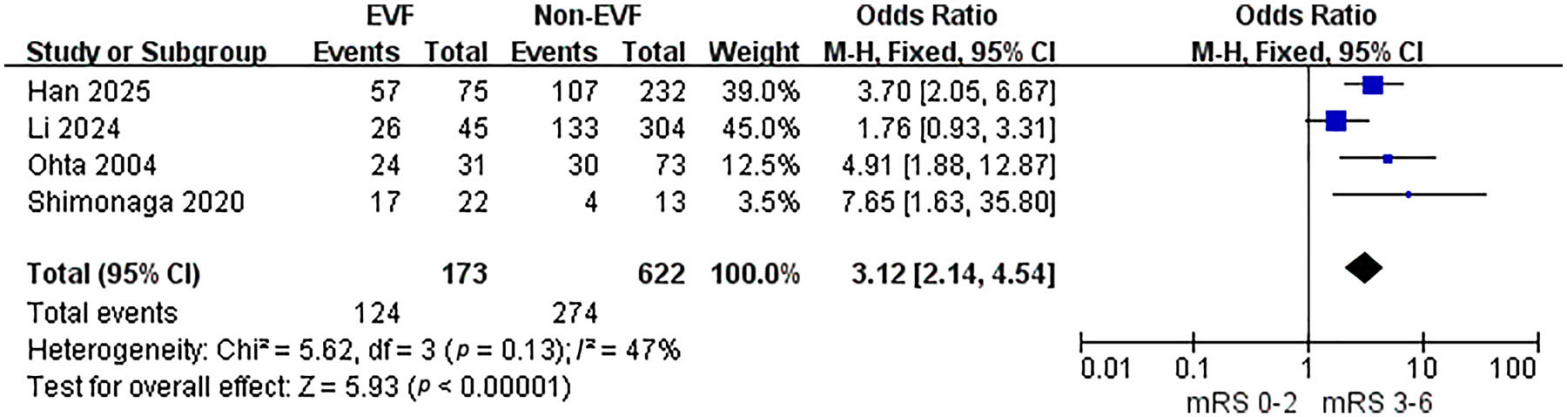
The association between EVF and poor prognosis.

To evaluate the relationship between EVF and poor prognosis (mRS > 2), a meta‐analysis of 4 included studies was performed using a fixed‐effect model. The analysis detected heterogeneity among the studies (*p* = 0.13, *I* ^2^ = 47%) (Figure 6). The results demonstrated that the presence of EVF was associated with a higher rate of poor prognosis at 90‐day follow‐up (OR = 3.12, 95% CI: 2.14–4.54, *p* < 0.001).

### 3.6 Sensitivity Analysis

The stability and sensitivity of the included studies were tested by altering the effects model. After the model modification, the majority of the subgroup studies retained their statistical significance, and the overall conclusions remained consistent. This consistency strongly indicated the stability of the results of this meta‐analysis. When the random‐effects model was switched to a fixed‐effect model, the results revealed that EVF was still associated with a higher rate of sICH after EVT (OR = 5.64, 95% CI: 3.71–8.58, *p* < 0.001). Conversely, when the fixed‐effect model was changed to a random‐effects model, the results showed that EVF was also associated with a higher rate of HT after EVT (OR = 4.81, 95% CI: 3.30–7.00, *p* < 0.001), a higher rate of PH after EVT (OR = 4.31, 95% CI: 2.12–8.73, *p* < 0.001), a higher rate of poor prognosis at 90 days (OR = 3.33, 95% CI: 1.89–5.87, *p* < 0.001), and a relatively high rate of HI after EVT (OR = 1.71, 95% CI: 0.78–3.77, *p* = 0.18).

We further performed a leave‐one‐out sensitivity analysis to evaluate the association between EVF and HT. The results demonstrated that the overall combined ORs spanned from 4.16 (95% CI: 3.13–5.52) to 4.88 (95% CI: 3.70–6.43), indicating that the current findings are robust and not substantially affected by any individual study.

### 3.7 Analysis of Publication Bias

**FIGURE 7:**
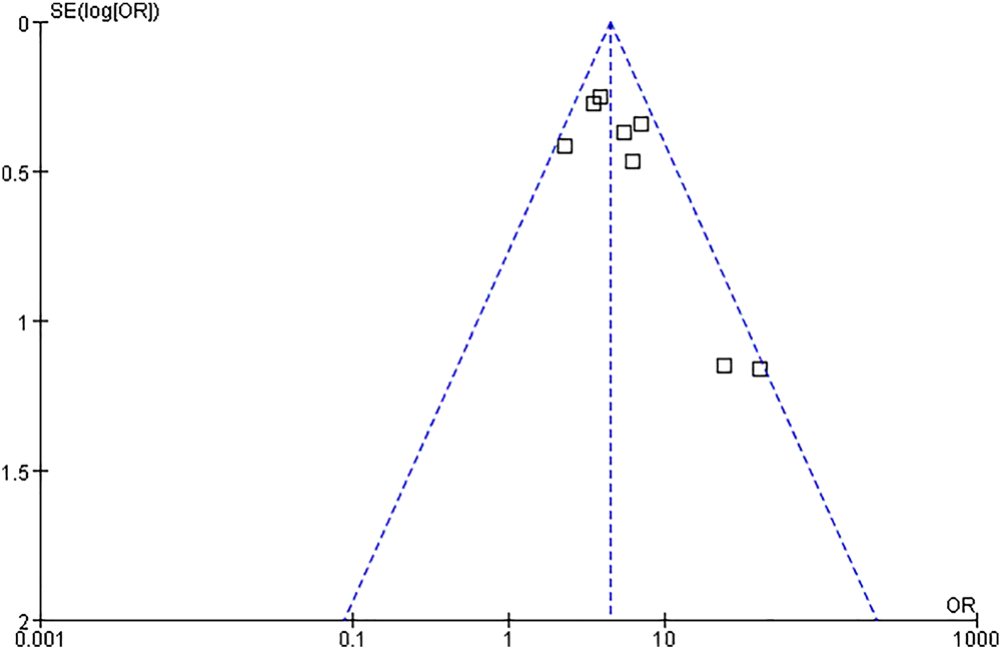
Funnel plots of publication bias for HT.

**FIGURE 8:**
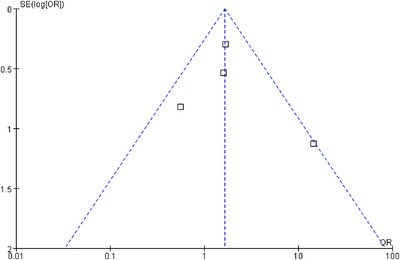
Funnel plots of publication bias for HI.

**FIGURE 9:**
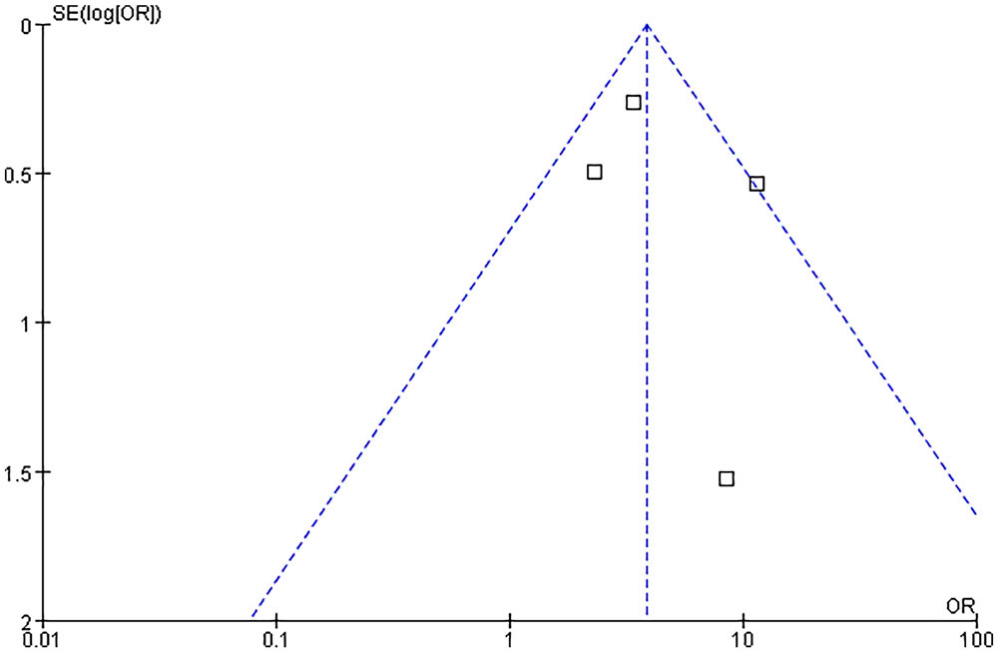
Funnel plots of publication bias for PH.

**FIGURE 10:**
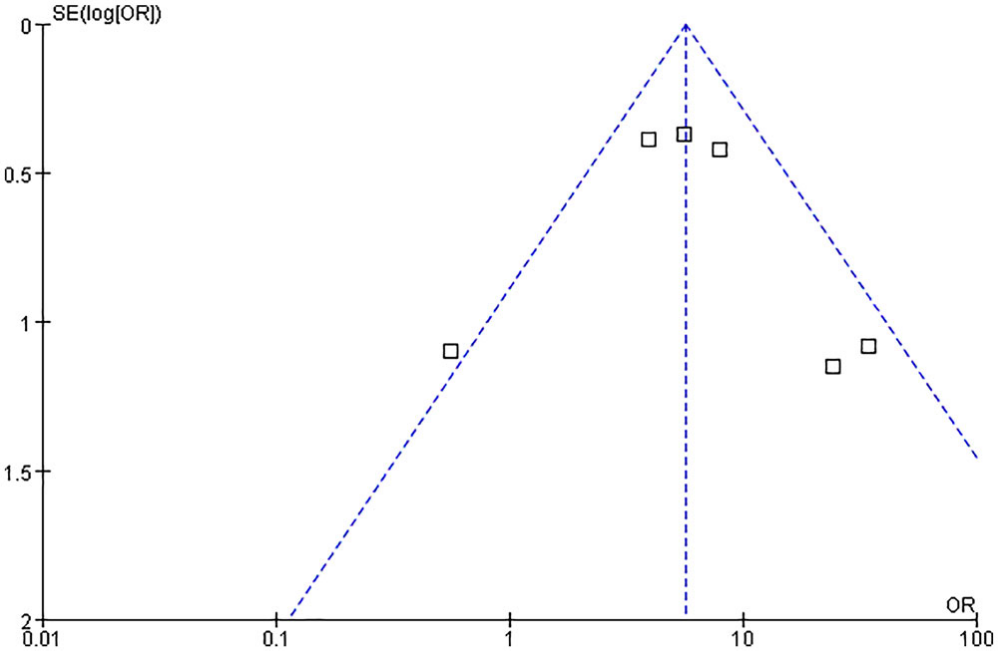
Funnel plots of publication bias for sICH.

**FIGURE 11:**
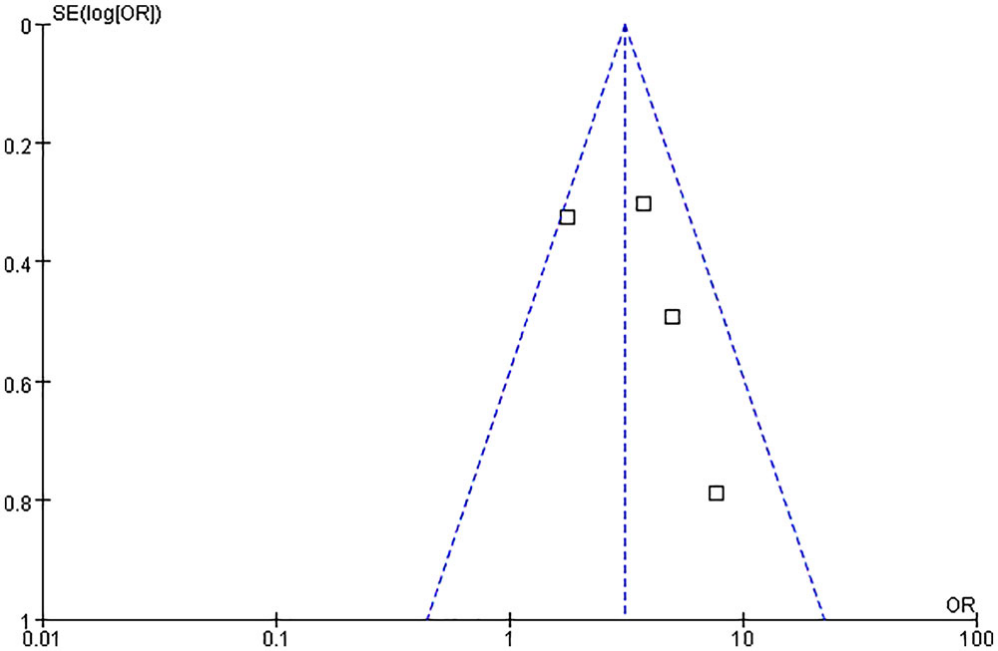
Funnel plots of publication bias for poor prognosis.

Funnel plots were employed to detect potential publication bias. The results of the funnel plot analysis for all endpoints demonstrated a relatively symmetrical distribution of the included studies (Figures 7, 8, 9, 10, 11).

## 4 Discussion

EVF represents a distinct angiographic entity that can be accurately identified using DSA. However, current researches on the associations between EVF and HT have primarily been limited to small‐scale retrospective cohort studies. There is a lack of comprehensive meta‐analyses. Therefore, our study aims to explore the relationship between EVF and HT through a meta‐analysis. Overall, the results of this meta‐analysis indicate that EVF is associated with an increased incidence of HT (OR = 4.5, 95% CI: 3.47–5.85, *p* < 0.001) and poor outcomes (OR = 3.12, 95% CI: 2.14–4.54, *p* < 0.001) after EVT. The results of sensitivity analyses demonstrated the robustness of our findings.

At the inception of EVT, arterial thrombolysis was the principal treatment option. EVF not only indicated irreversible cerebral infarction but was also associated with a markedly higher incidence of HT. This was due to EVF disrupting normal cerebral vascular dynamics, making patients more vulnerable to HT during arterial thrombolysis (Ohta et al. 2004). In subsequent developments, mechanical thrombectomy has become the mainstay of EVT. Of the 1758 patients enrolled in our study, 462 (26%) had EVF after EVT. Among EVF‐positive patients, 47.6% (220/462) experienced HT. The observed proportion of HT in EVF‐positive patients is in line with the results of previous investigations, suggesting a stable relationship between EVF and HT incidence after EVT (Han et al. 2025; Ohta et al. 2004; Cartmell et al. 2018; Shimonaga et al. 2020; Elands et al. 2021; Janvier et al. 2022; Yu et al. 2023; Li et al. 2024). In recent 5 years, the relationship was further identified by many studies. Elands et al. (2021) demonstrated that EVF on angiographic imaging was a potential predictor of reperfusion hemorrhage. Yu et al. (2023) found EVF was independently associated with HT and PH after EVT, especially in patients with severe stroke. Li et al. (2024) found that EVF was independently associated with ICH, sICH, and malignant cerebral edema after successful recanalization of EVT. Compared to the non‐EVF group, the EVF group had a significantly higher incidence of ICH (66.7% vs. 22%), sICH (28.9% vs. 4.9%), and malignant cerebral edema (20% vs. 6.9%). Notably, patients with cortical artery‐to‐cortical vein (Type I) EVF had a higher mortality rate than those with lenticulostriate artery‐to‐thalamostriate vein (Type II) EVF. Although statistical significance was not established, early filling of the thalamostriate vein may pose a higher risk of sICH compared to early filling of the cortical vein (Ohta et al. 2004). Janvier et al. (2022) found that EVF after complete successful recanalization in AIS was an independent predictor of sICH and integrated EVF into a risk score of sICH prediction. Time‐Alberta Stroke Program Early CT‐Glycemia‐EVT score is a simple tool with readily available clinical variables with good performances for sICH prediction after EVT. Han et al. (2025) established EVF as an independent risk factor for HT and unfavorable outcomes after EVT. As we all know, HT is closely associated with clinical prognosis. Compared with the non‐EVF group, the EVF group was more likely to experience worse clinical outcomes (Han et al. 2025; Ohta et al. 2004; Shimonaga et al. 2020; Li et al. 2024).

EVF serves as a valuable clinical marker with far‐reaching implications in the medical field. First and foremost, it can act as an early warning sign for irreversible cerebral infarction. By detecting EVF, stroke physicians can intervene promptly, potentially saving patients from further neurological damage. Moreover, EVF is closely associated with an increased incidence of HT. Understanding this relationship enables clinicians to better assess the risks associated with EVT. For example, if a patient with EVF is scheduled for arterial thrombolysis, the medical team can be more cautious, closely monitor the patient, and adjust treatment plans to minimize the risk of HT. In addition, as a clinical marker, EVF helps in prognostic evaluation. Patients with EVF are more likely to experience poor clinical outcomes. This knowledge allows healthcare providers to communicate more effectively with patients and their families, setting realistic expectations and planning appropriate long‐term care. Overall, EVF provides crucial insights for both diagnosis and treatment, highlighting its importance in improving patient management and potentially enhancing overall outcomes in cerebrovascular diseases.

Our study has several significant limitations. Most of the studies included in this analysis were single‐center, retrospective, small‐sample cohort investigations. The lack of prospective, multicenter, randomized double‐blind, large‐sample cohort studies means there are substantial risks of bias. Both design flaws and potential publication biases may have distorted the results, thus compromising the generalizability of our findings. We should acknowledge that the meta‐analysis results in subgroup analysis (the association between EVF and HI/PH) are significantly influenced by Yu et al., 2023 due to its high weight, and discuss its potential impact on the findings, such as introducing bias or affecting the stability of results. In some studies, once EVF was detected during angiography, subsequent EVT was stopped. This could underestimate the risk of HT in the EVF group, masking the true relationship between EVF and HT and skewing the overall results. Some studies included cases of posterior circulation. Since these cases only evaluated the premature appearance of veins from cortical arteries to cortical veins, and the HT incidence varies between the posterior and anterior circulations, their inclusion may have confounded the outcomes. There was also heterogeneity in treatment methods across the included studies. Some studies used only arterial thrombolysis while others mainly adopted mechanical thrombectomy. This variability likely influenced the occurrence of HT, complicating data interpretation. In addition, no subgroup analysis was conducted to explore the associations between different types of EVF and HT and their impact on prognosis. Future research should identify which type of EVF is more likely to lead to sICH and an unfavorable outcome.

## Author Contributions

**Jiayu You**: conceptualization, investigation, funding acquisition, writing – original draft, methodology, validation, visualization, writing – review and editing. **Xingqiang Li**: software, formal analysis, project administration, data curation, supervision, resources.

## Ethics Statement

The authors have nothing to report.

## Consent

This meta‐analysis utilized publicly available data from previously published studies. Individual participant consent was not required, as the research does not involve direct interaction with human subjects or identifiable data. All included studies were reviewed for their original consent procedures, and it was confirmed that the primary studies obtained informed consent from participants in accordance with ethical guidelines.

## Conflicts of Interest

The authors declare no conflicts of interest.

## Peer Review

The peer review history for this article is available at https://publons.com/publon/10.1002/brb3.70663

## Supporting information




**Supplementary Material**: brb370663‐sup‐0001‐SuppMat.docx

## Data Availability

This study is a meta‐analysis of publicly available data and does not involve direct interaction with human subjects or the use of identifiable information.
