# Supplementary material for: A Meta‐Analysis of the Association Between Early Venous Filling and Hemorrhagic Transformation After Endovascular Treatment in Acute Large Vessel Occlusion
Source: Brain Behav. 2025 Jul 7;15(7):e70663. doi: 10.1002/brb3.70663 (PMC12230624; doi:10.1002/brb3.70663)
Supplement: Supplementary file 1 — Supplementary Material: brb370663‐sup‐0001‐SuppMat.docx [file BRB3-15-e70663-s001.docx]

Search formulas of Past Systematic Review;

Database:

PubMed

Date of search:

March 20^th^, 2025

Search strategy:

((("Early Venous Filling"[Mesh]) OR "EVF"[All Fields] OR "Early Venous Filling Sign"[All Fields] OR "Early Appearance of Venous"[All Fields] OR "Premature Venous Filling"[All Fields])

AND (("Hemorrhagic Transformation"[Mesh]) OR "HT"[All Fields] OR "Bleeding Transformation"[All Fields] OR "Intracerebral Hemorrhage Transformation"[All Fields]))

AND (("Acute Ischemic Stroke"[Mesh]) OR "AIS"[All Fields] OR "Acute Cerebral Ischemia"[All Fields] OR "Brain Ischemic Attack, Acute"[All Fields]))

AND (("Endovascular Treatment"[Mesh]) OR "EVT"[All Fields] OR "Endovascular Intervention"[All Fields] OR "Intracranial Endovascular Therapy"[All Fields]))
